# Gas Detection Using Portable Deep-UV Absorption Spectrophotometry: A Review

**DOI:** 10.3390/s19235210

**Published:** 2019-11-28

**Authors:** Sulaiman Khan, David Newport, Stéphane Le Calvé

**Affiliations:** 1 School of Engineering, Bernal Institute, University of Limerick, Limerick V94 T9PX, Ireland; sulaiman.khan@ul.ie (S.K.); david.newport@ul.ie (D.N.); 2 Université de Strasbourg, CNRS, ICPEES UMR 7515, F-67000 Strasbourg, France; 3 In’Air Solutions, 67087 Strasbourg, France

**Keywords:** deep ultraviolet, absorption spectrophotometry, spectroscopy, ultraviolet light sources, LEDs, hollow core waveguides, photodiodes, ozone, SO_2_, NO_2_, VOC, BTEX

## Abstract

Several gas molecules of environmental and domestic significance exhibit a strong deep-UV absorption. Therefore, a sensitive and a selective gas detector based on this unique molecular property (i.e., absorption at a specific wavelength) can be developed using deep-UV absorption spectrophotometry. UV absorption spectrometry provides a highly sensitive, reliable, self-referenced, and selective approach for gas sensing. This review article addresses the recent progress in the application of deep-UV absorption for gas sensing owing to its inherent features and tremendous potentials. Applications, advancements, and challenges related to UV emission sources, gas cells, and UV photodetectors are assessed and compared. We present the relevant theoretical aspects and challenges associated with the development of portable sensitive spectrophotometer. Finally, the applications of UV absorption spectrometry for ozone, NO_2_, SO_2_, and aromatic organic compounds during the last decades are discussed and compared. A portable UV absorption spectrophotometer can be developed by using LEDs, hollow core waveguides (HCW), and UV photodetectors (i.e., photodiodes). LED provides a portable UV emission source with low power input, low-intensity drifts, low cost, and ease of alignment. It is a quasi-chromatic UV source and covers the absorption band of molecules without optical filters for absorbance measurement of a target analyte. HCWs can be applied as a miniature gas cell for guiding UV radiation for measurement of low gas concentrations. Photodiodes, on the other hand, offer a portable UV photodetector with excellent spectral selectivity with visible rejection, minimal dark current, linearity, and resistance against UV-aging.

## 1. Introduction

Gas detection has become an integral part of today’s industrial and domestic life. It has several applications, ranging from environmental sensing in the stratosphere to indoor air quality and even diagnosis of different diseases in human bodies. Some common applications of gas sensors are in the detection of toxic and flammable gases for health and safety, detection of key species in food industry, monitoring of indoor air quality and exhaust of harmful gases from fossil fuel burning, monitoring of greenhouse gases for environmental protection, and diagnostics of different diseases by identifying potential biomarkers such as volatile organic compounds (VOCs) [1,2,3,4,5,6]. Usually, quantitative detection of gases is performed by laboratory analytical devices such as gas chromatography with a flame ionization detector, which preclude portability and real-time observation. Currently, there is a demand for portable gas analyzers to acquire analytical data in real-time with high sensitivity, low pure gas consumption (i.e., carrier gas), and low power consumption. Typical gas sensing techniques are electrochemical [7], semi-conductor [8], photo-ionization detection (PID) [9,10], piezoelectric [11,12] and optical technique [13,14,15,16]. Each technique has its advantages and limitations, as summarized in Table 1. 

**Figure 1:**
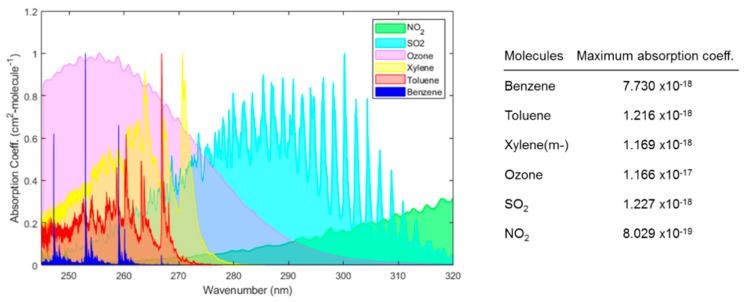
The ultraviolet (UV) absorption spectrum of different gases. The absorbance of each gas is normalized by the respective maximum absorption coefficient of the gas. The data of each molecule were obtained from HITRAN [18] and then plotted on a wavelength scale.

Optical gas sensors based on absorption have been shown to provide a sensitive and selective approach with minimal drift, rapid time response, and a low cross-response to other gases [9,14]. The measurement is self-referenced and reliable as the transduction is based on direct measurement of a molecule’s physical property (i.e., absorbance at a specific wavelength). Real-time data are obtained without changing the nature of the gases, which can be critical for process control [17]. There are several gas molecules that absorb strongly in ultraviolet (UV) (wavelength range, 180 nm to 400 nm) and deep-UV (wavelength range, 180 nm to 280 nm), thus by exploiting the absorption property of these compounds, a sensitive and selective sensing platform can be developed. The latest advancements in semiconductor and optoelectronics, for instance, LED and photodetectors and optofluidics have given an opportunity to develop miniaturized UV spectrophotometers for gas sensing applications. For example, molecules of atmospheric interest such as ozone, NO_2_, SO_2_, H_2_S, benzene, toluene, ethylbenzene, and xylene (-p, -m, -o) exhibit strong absorption bands in the deep UV region (λ < 300 nm) as shown in Figure 1. 

Recently, there has been growing interest in the application of deep UV spectrophotometry for gas sensing application. Therefore, this paper addresses the progress made in the UV spectrophotometry and its application for different gases. In the first section, a brief overview of UV spectrophotometry is provided. In the following sections, each component of UV spectrophotometer is discussed with the latest developments and challenges. The research conducted during the last decades in the domain of deep-UV spectrophotometry for different gases is described and compared by using various analytical parameters. In the final section, the conclusion of the study is provided with a future outlook.

## 2. UV Spectrophotometry

UV absorption spectrometry is a well-known analytical technique and has been applied extensively in analytical chemistry, biochemistry, and biomedical applications due to its simplicity, flexibility, low cost, and convenience [19]. Absorption is an attenuation of electromagnetic radiations at a specific energy as it passes through an analyte. Electromagnetic radiation with high energy (lower wavelength) will cause excitation of an electron from lower energy state to high energy state. Absorption occurs when the energy of photons Δ*E* matches with the energy, *E*_2_*-E*_1_, between the two-energy level of the analyte. When an analyte absorbs UV/visible radiations, it mainly undergoes a change in its valence electronics configurations. UV absorption spectrometry is associated with the transition of electrons either from a bonding state (π) to an anti-bonding state (π*) or from a non-bonding state (n) to an anti-bonding state (π*) at a specific light energy. For example, for benzene, the transition from the bonding (π) to the anti-bonding state (π*) happens at radiation with energy 4.88 eV, which corresponds to a wavelength of 254 nm. Absorption is usually quantified using transmittance and absorbance. Transmittance represents the ratio of radiant power *I*, passing through a sample to that of power from the radiant source *I_o_* as given by $T=\frac{I}{I_{o}}$. Most of the detectors for absorption are based on measuring transmittance (T < 100%). The attenuation of radiation is alternatively expressed using absorbance *A*, which is defined by

(1) $$A=-\log\left(T\right)=-\log\left(\frac{I}{I_{o}}\right)=\log\left(\frac{I_{o}}{I}\right).$$

Absorbance, *A* and concentration, *c* (molecule cm^−3^) are related to each other according to the Beer–Lambert law as

(2) A=σ l c 

where *l* represents the optical length of the gas cell in cm and *σ* represents the absorption cross-section of a gas molecule in cm^2^-molecule^−1^. Its value depends on the wavelength and gives the probability that the analyte will absorb a photon of a given energy. This law is a limiting law and is valid for low concentrations of analyte and for strictly monochromatic radiation, provided that the physical and chemical properties of absorbing species do not change with the concentration. 

In absorption spectrophotometry, long optical path length and strong absorption cross-sections are generally associated with high sensitivity. However, the sensor saturation may occur, which may limit the sensitivity and linearity. Marcus et al. [20] investigated the sensor saturation and derived equations based on Beer–Lambert law for optimized transmittance, optical path length, and absorption cross-section for a specific range of ozone concentration to prevent sensor saturation. The optimized optical path length, *l_op_* and optimized cross-section *σ_op_* for ozone concentrations *c*_1_ and *c*_2_ were derived as expressed as,

(3) $$l_{op}=\frac{10^{6}\;R\;T\;(\ln c_{1}-\ln c_{2})}{\sigma \;N_{A}P\;\left(c_{1}-c_{2}\right)};\;\sigma_{op}=\frac{10^{6}\;R\;T\;(\ln c_{1}-\ln c_{2})}{l\;N_{A}P\;\left(c_{1}-c_{2}\right)}$$

whereas *c* is the concentration of an analyte in ppm; *σ* is absorption cross-section in m^2^-molecule^−1^; *T* is absolute temperature in *K*; *P* is pressure in atm; R is ideal gas constant, 8.205746 × 10^−5^ atm-m^3^ mol^−1^- K^−1^; N_A_ is Avogadro’s constant, 6.02214199 × 10^23^ molecule-mol^−1^. Transmittance, *T* of light affects the sensitivity of the measurement. Marcus et al. [21] derived optimized transmittance equation for highly sensitive measurement and was validated numerically and experimentally as given by,

(4) $$\frac{dT_{r}}{dc}=\;\frac{T_{r}\ln T_{r}}{c}\;T_{r}=\exp\left(\frac{-\sigma \;N_{A}\;P\;l\;c}{10^{6}\;RT}\right).$$

The Beer–Lambert equation can be expressed in its integral form where *x* is the variable length as given as [22],

$$I=I_{o}\;e^{-\sigma xc}$$

(5) $$\frac{dI}{dx}=-\sigma \;c\;I_{o}e^{-\sigma xc}\;\mathrm{also}\;\frac{dI}{dc}=-\sigma \;x\;I_{o}e^{-\sigma xc}.$$

**Figure 2:**
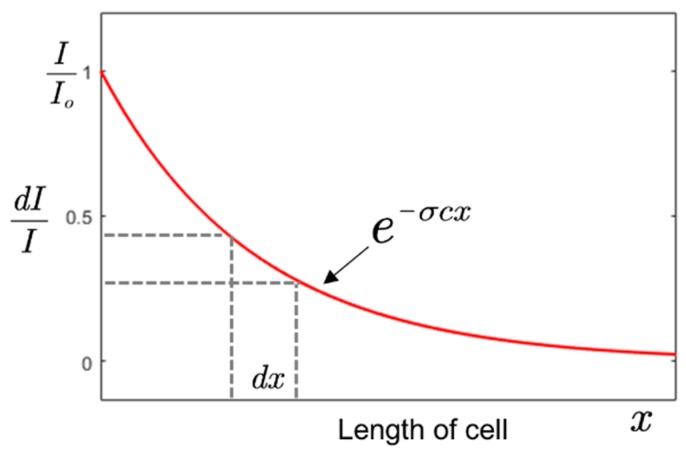
Variation of relative intensity with the length of a gas cell.

According to Equation (5), a sensor with a high concentration resolution at a shorter length can be realized by using gas with a high absorption cross-section or/and by employing UV source with a high spectral power density (*I_o_*) close to the maximum absorption power of an analyte. The differential variation of intensity with the length of the gas cell is shown in Figure 2. 

**Figure 3:**
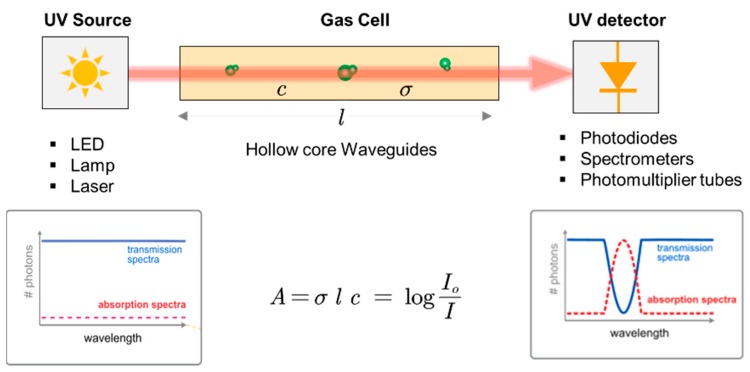
Schematics of UV absorption spectrophotometry. The plots on the left and right represent the signal from the emission source and the final signal recorded at the photodetector after passing through the gas cell, respectively.

A typical UV-spectrophotometer is mainly composed of a UV emission source, gas cell, and UV photodetector, as summarized in Figure 3, with its working principle. Each component of the spectrophotometer is discussed in the following sections. 

## 3. UV Sources

### 3.1. General Overview

**Figure 4:**
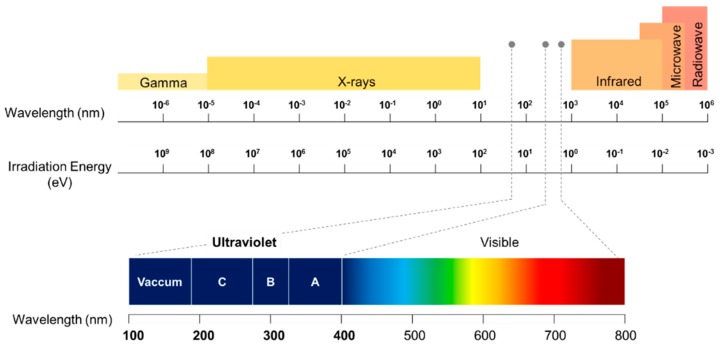
A spectrum of electromagnetic radiation. The spectral range of different UV region is represented.

UV is a component of the electromagnetic radiation spectrum with a wavelength from approximately 180 nm to 400 nm, as shown in Figure 4. UV radiation can be sub-divided into further spectral bands as:

UV-A (near UV), 315–400 nm.

UV-B (middle UV), 280–315 nm.

UV-C (far UV), 180–280 nm.

UV-A can easily transmit through air and glass. On the other hand, UV-B and UV-C are transmitted through air and quartz but absorbed by the glass. UV with a wavelength less than 280 nm (i.e., UV-C) are usually called deep-UV. Radiations from 180 nm to 10 nm are called vacuum or extreme UV as these radiations only propagate in a vacuum.

**Figure 5:**
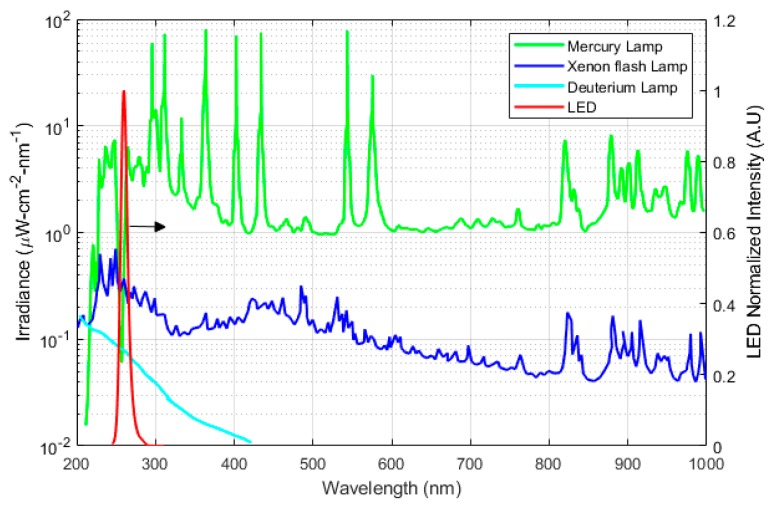
The output intensity of different UV sources. LED (80 µW, Mightex System US) data were obtained from Khan et al. [24] and other data were sourced from Hamamatsu Photonics. Mercury Xenon lamp (L2423, 200 W), Xenon flash lamp (L11957, 20 W), Deuterium Lamp (L9519, 30 W) [25].

Common UV sources are open arcs, fluorescent and incandescent sources, lasers, and LEDs. UV sources are characterized using wavelength range, optical output stability, lifetime, and input power. In spectroscopy applications, a UV emission source should be stable and have enough power intensity for the desired wavelength. Light stability is commonly characterized by fluctuation (short-term stability) and drift (long-term stability). The stability of the emitted light output is usually quantified by the ratio of variation in the intensity of emitted light to the mean intensity of the emitted light. It is an essential factor in defining the accuracy and reliability of a spectrophotometer. Typically for analytical detection, the concentration is determined with sequential measurements of the sample and reference gases that are alternatively injected into the gas cell. It is therefore important to have a stable light output between the two measurements. Lifetime is usually defined as the time when the light output exceeds a specified range of fluctuations [23]. The emission spectra of the common UV sources, i.e., xenon flash lamp, deuterium lamp, mercury lamp, and UV LED, are shown in Figure 5. Lifetime affects the cost of equipment. Shorter lifetime results in frequent replacement impacting the total price of use.

Deuterium lamps are relatively more stable (fluctuation < 0.005%) than other UV lamp alternatives. It has relativity high-intensity light output with longer lifetime. However, the major shortcomings of deuterium lamps are size, a highly stable power supply (150 W) requirement, and a warm-up time of approximately 30 min for thermal equilibrium. Low-pressure mercury lamps have been traditionally applied for spectrophotometric detection of ozone and Benzene, Toluene, Ethylbenzene, and Xylenes (BTEX) as its emission spectrum (i.e., ~254 nm) matches strongly with the absorption spectra of these molecules. The Mercury Xenon lamp has also been used as a UV emission source in spectrophotometric studies. Its limitations are that it requires high power input and has low output stability. Also, mercury lamps contain hazardous waste. There is a pressing need of reducing the mercury utilization by different agreement and regulations such as Minamata Convention on Mercury, which is an international treaty for elimination of mercury usage with its implementation by 2020 [26]. In comparison with other UV sources, LEDs are an attractive option for future spectrophotometric devices owing to portability, low cost, and low power consumption. A comparison of different UV sources is summarized in Table 2.

**Figure 6:**
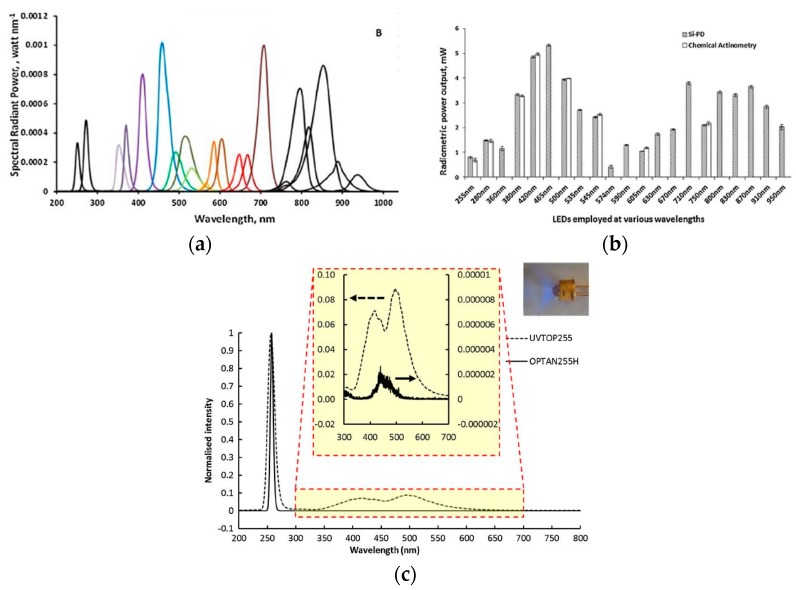
(**a**) The emission spectrum of different LEDs with different peaks [32]. (**b**)The power output of various LEDs at different wavelength [32]. (**c**) Output UV spectrum with parasitic emissions for aluminum nitride (AlN)-based LEDs (dashed lines) and sapphire-based LEDs (solid lines) [23]. Adapted with permission from Elsevier.

LEDs have been successfully applied in analytical chemistry [27,28,29] and led to the development of affordable, cost-effective, and compact instrument designs with low power consumption. Bui et al. [30], Dasgupta et al. [31], and Yan Li et al. [23] have reviewed the development and application of LED in analytical sciences. For details, the interested readers are invited to refer to these articles. LEDs are solid-state semiconductor diodes, which emit light due to the recombination of hole-electrons pair. The energy of an emitted photon depends on the bandgap of the semiconductor. LEDs are small (emitter chip size for 5 mm is 250 µm^2^) and portable with low optical noise, which can be easily incorporated into flow-through detectors, for instance, as absorbance measurement detectors. LEDs have lower intensity drifts, low cost, long lifetime (10^4^ h), and low heat generation [31]. They can be operated in pulse mode up to GHz range for applications with fast switching, which is not possible with the conventional UV sources. These features made them a suitable candidate for incorporation into low-power portable devices. They are quasi-monochromatic sources that have typically narrow emission band less than 30 nm in width. The narrow emission bandwidth of LED covers the absorption band of molecules without monochromators for absorbance measurement of a target analyte. As can be seen from Figure 1, the absorption spectra of most molecules can be covered with a bandwidth of 30 nm. To broaden the emission band, an array of LEDs can be used to cover a wide range of absorption spectra. The available range of LEDs is usually from 250 to 900 nm, as shown in Figure 6. LEDs with lower wavelength (λ < 300 nm) have lower power efficiency, low power output (1 W), limited wavelength range, and high price tags [32]. Owing to its size and shape, LEDs give more degree of robustness and miniaturization compare to the conventional light UV sources. Different variants of LEDs are available such as surface mount, high power version (for higher wavelengths >350 nm), and LEDs with various integrated lens covers. The top cover of LED, which acts as a lens, can be modified for desired applications. For analytical applications, usually a collimated or focused beam is needed, which can be achieved by modifying the shape of the LED headcover. For example, a ball lens is a good option to realize a tight focusing of light. By changing the curvature of the LED cover to high-radius curvature (linearized profile) by careful cutting and polishing, a collimated UV beam can be achieved, which can be then easily coupled with optical fibers. The total cost of typical ozone monitors using a UV-C LED (OPTAN 255, 1 mW) as a source is estimated to be $350 (the total cost is sum of the instrument cost (emission source, optical filter, power supply, detector, and heat enclosure) and operating cost (i.e., power consumption, lamps replacement, and disposal)). On the other hand, a rival detector based on UV mercury lamp (TUV 11 W, mercury lamp) is around $580. The lower cost of LED-based monitor is mainly due to the absence of optical filters and heat enclosure and lower operating and maintenance cost [33]. 

### 3.2. Challenges and the Latest Advancement

Several LED-based analytical devices have been developed for infrared and visible light application. However, LEDs for lower wavelength (λ < 350 nm) need a higher bandgap at the semiconductor junction. At lower wavelength, the photon conversion efficiency is small, which lead to low-power UV emission, high cost of devices, and negative thermal effects [34]. Presently, LEDs based on AlGaN semiconductor can emit light with wavelength down to 240 nm, but there is a growing demand for a stable and high-power LED with emission wavelength lower than 200 nm for sterilization and sensing applications. 

LED is a non-linear electronics device and requires a constant-current supply for operation. The constant current is usually supplied by using a high voltage with a current limiting resistor or by employing an active current control. Active control current sources are less susceptible to temperature changes [30]. The optical output of an LED is sensitive to the junction temperature. The optical power decreases with the increase in junction temperature. For deep-UV LEDs, proper thermal management should be considered for a stable LED output. Another approach for minimizing the drift due to the temperature is by employing a self-reference approach in parallel. The typical power output for UV-C LED is less than 1 mW, which is relatively small for transmission in the gas cell. The summary of different UV LEDs power output is summarized in Figure 6a,b.

UV LEDs show parasitic broadband emission in the visible region, which should be taken into account when designing an absorption-based detector for UV applications [35]. A typical example of parasitic emission in the output spectrum of UV-C LED is shown in Figure 6c [23]. Parasitic emission is dominant at a low forward current (<5 mA) [36] and is predominantly caused by the presence of undesirable bands of lower energy bands in the emitting semiconductor [37,38,39] or the presence of fluorescence or phosphorescence contaminant in LED structure [40]. Recently, aluminum nitride-based (AlN) LEDs have been developed, which give high optical power out (1.5 mW, 100 mA) with a low value of parasitic emission in comparison with sapphire-based LEDs (30 mA). Li et al. [23] investigated the performances of an AlN based LED for analytical applications and reported that AlN based LEDs have minimal parasitic emission in comparison with the sapphire based LEDs as shown in Figure 6c. The ratios of parasitic emission to the desired UV emissions were 0.0002% and 8% for AlN and sapphire based LEDs, respectively.

UV LEDs offers spatial stability, intensity stability, and narrow emission band with low optical losses, which can be used to achieve better limits of detection than the conventional sources. By minimizing power supply noise, temperature fluctuation, and mechanical stability of optics, the performance can surpass the traditional UV lamp sources [41,42,43].

## 4. Optical Gas Cell

### 4.1. General Overview

An optical gas cell (OGC) is a platform where the photons (UV radiation) and gas analyte molecules interact. According to the Beer–Lambert relationship, the sensitivity of a spectrophotometer for a specific gas is defined by the length of the gas cell. There is a trade-off between sensitivity (i.e., the longer optical path of OGC) and portability of the spectrophotometer for a specific gas. Different types of gas cell have been developed to achieve longer optical paths such as single-pass gas cells, multiple-pass gas cells, and resonant cavity. Multiple-pass gas cells (e.g., Herriot or white) are bulky and require a high volume of gas, thus have limited time resolutions. Single-pass cells, on the other hand, have low-volume, ease of integration, and fabrication. 

**Figure 7:**
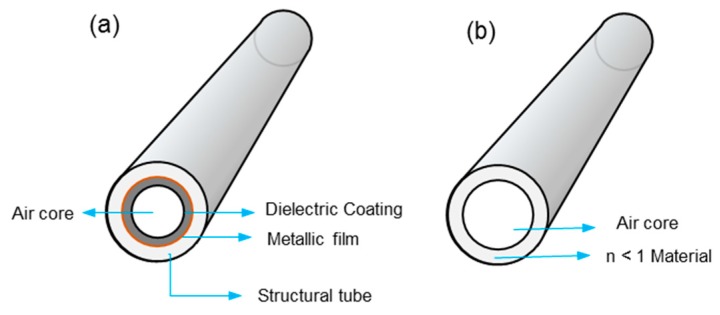
(**a**) Leaky mode hollow core waveguides (HCW) and (**b**) Attenuated total-reflection (ATR) HCW.

Hollow core waveguides (HCWs) provide an attractive option to be employed as a single-pass gas cell for UV absorption spectrophotometry. HCWs were initially designed as a light-tube for the transmission of high-peak-power laser light for industrial and medical applications [44,45]. They have been applied for the detection of different analytes in liquid or gas for environmental, process monitoring, and biomedical applications [46,47]. HCW acts as a light-pipes by transporting radiation inside the co-axial hollow core of the fiber. It can be applied as a miniature gas cell for guiding the radiation for measurement of low gas concentrations. Depending on the guiding mechanism, HCWs can be classified into attenuated total-reflection (ATR) or leaky-mode (LM) waveguides. ATR waveguides are made of tubes with refractive index (*n*) less than that of the air-core (*n* = 1) whereas LM waveguides are composed of a tube, with inner wall coated with materials of refractive index greater than one (*n*>1) as shown in Figure 7 [48]. In ATRs, the walls are made of alternating dielectric layers, exploiting the photonic bandgap, which gives a frequency-dependent refractive index contrast [49]. The dielectric layer acts as an optical stop band in the radial direction, and the radiation is propagated only in the axial direction for a selected range of frequencies [50]. The LM waveguides are composed of structural tubes of silica, metal, sapphire, or plastics with a reflective coating on the interior walls of the hollow core. The radiation is propagated by metallic reflection inside the coaxial hollow core. Some of the common reflective metals for the propagation of radiation, from mid-IR to deep UV are silver, gold, aluminum, and dielectrics. In this type of waveguide, the metallic coating is often covered with dielectric coating for chemical inertness of the coating. The coating is applied using chemical vapor deposition or liquid coating methods [51].

ATRs have limited applications due to the properties (refractive index, absorption band, etc.) of compatibility of material for different frequencies and offer a narrow transparent window. LM waveguide, on the other hand, can transmit broadband of wavelength, from mid-IR to deep UV radiations and have relatively simple fabrication process. These types of HCWs have been applied as IR gas cells for detection of several gases. For instance, Kim et al. used silver/silver-halide coated HCW for detection of industrial relevant gases, i.e., CO_2_, CH_4_, and C_2_H_5_Cl and their mixture [52]. Leaky mode HCWs have been applied for sensing of carbon monoxide [53], dimethyl sulphide [54], and methane [55].

There is a demand for waveguides for a number of UV applications, for instance, spectrophotometry and UV exposure in microfabrication. Optical fiber technology for UV transmission is not as developed as for IR applications due to the incompatibility of different materials with UV. For example, glass and silica-based materials are not transparent at low wavelength and materials such as fluorides are not suited to optical fiber production. Due to these challenges, LM waveguides offer a suitable and simpler option for UV absorption spectrometry. In such waveguides, the optical transmission depends upon the metallic or dielectric coating deposited on the inner wall of the metal, polymer, or glass tubing. In addition to that, the size and geometry of the HCW affect the efficiency of light transmission. HCWs with smaller diameters exhibit higher attenuation [56],

(6) $$\alpha \;\left[\frac{dB}{m}\right]\;\propto \frac{1}{a^{3}}$$

where *α* represents the attenuation in dB-m^−1^ and *a* represents the inner radius of HCW. The orientation of the HCW also affect the attenuation as the optical losses *L_HCW_* increases with the bending radius (*R*) or curvature *(C =* 1/*R*) of the HCW,

(7) $$L_{HCW}\left[dB\right]\propto C.$$

The coupling efficiency is also critical and is related to maximum acceptance angle, i.e., *N.A = Sin(θ_max_)* where *N.A* is the numerical aperture. Therefore, for the design of an efficient portable gas sensing system, the trade-off between the optical efficiency, size, and geometry of the HCW must be considered. For establishing a constant pressure drop across the HCW, a pressure controller is usually employed, for example a forward-pressure controller installed at the end of HCW is a suitable option for easier fluidic connections.

**Figure 8:**
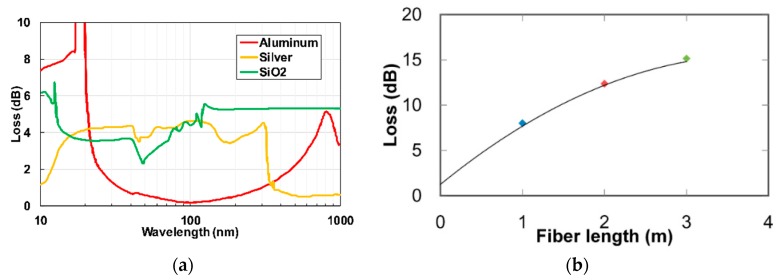
(**a**) Optical losses for glass HCW with inner walls coated with aluminium (red), silver (yellow) and SiO_2_ (green) [57]. (**b**) Optical losses of glass HCW coated with aluminum for different length of HCW [58].

Aluminum-based ATR HCW exhibited excellent low transmissions losses in the UV to near infrared radiations (i.e., 200 to 800 nm). Matsuura et al. [57] developed a leaky mode hollow-core optical fiber for UV and vacuum UV applications. Different coating materials were characterized, as shown in Figure 8a. The aluminum-coated fiber showed low losses compared to silver in the UV range of wavelengths. A transmission-loss spectrum of the aluminum HCW with an inner diameter of 1 mm for different length is shown in Figure 8b [58]. It can be observed from the figure that losses do not change linearly with the HCW length because there exist low-order modes and attenuation of high-order mode in long fibers.

The compatibility of the coating material of the HCW with the gas mixture can also influence optical transmission performance. For example, when using aluminum HCW, humidity should be well-controlled prior to flushing the gas. Water molecules are adsorbed on the surface of the aluminum, thereby changes the reflectivity of the surface. In order to address this issue, the instalment of Nafion tubing to trap humidity prior the gas stream injection into the detection cell and quartz lining of the aluminum tubes avoiding any surface oxidation can minimize the effect of humidity [59].

### 4.2. Challenges and the Latest Advancement

**Figure 9:**
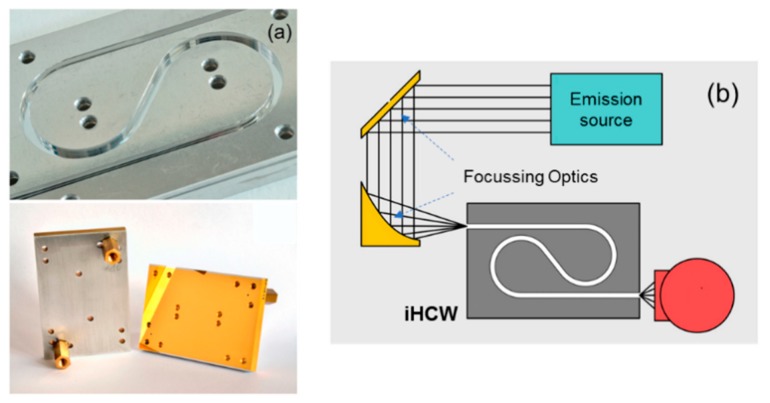
(**a**) i-HCW developed on the aluminum substrate [65]. (**b**) Setup gas detection with substrate-integrated hollow-core waveguides (i-HCW) with IR emission source and detector. Adapted with permission from [65], copyright 2019 American Chemical Society.

For a compact IR spectrophotometer, substrate-integrated hollow-core waveguides (i-HCW) have been developed to achieve longer optical path lengths on a small footprint area. i-HCWs are layered structures integrated with a solid-state material providing a light propagating channel in a compact volume as shown in Figure 9. i-HCW can be tailored according to a specific gas requirement and can be fabricated using cost-effective processes such as hot embossing or 3D printing. This type of HCWs (i.e., i-HCWs) have been applied for IR spectroscopy to detect several gases, for example NO_2_ [60], Ozone [61], methane [62,63], and isoprene [64]. Wilk et al. [65] demonstrated, such an HCW with 75 mm × 50 mm (L × W) to realize an optical path length of 22 cm. The device demonstrated a spectroscopic gas detection of butane, CO_2_, cyclopropane, isobutylene, and methane with limit of detection in the range of 6 to 11 ppm_v_. The application of iHCW for deep UV has not been reported, but this type of HCWs have a tremendous potential to be extended to deep UV absorption spectrometry by carefully considering the compatible materials.

**Figure 10:**
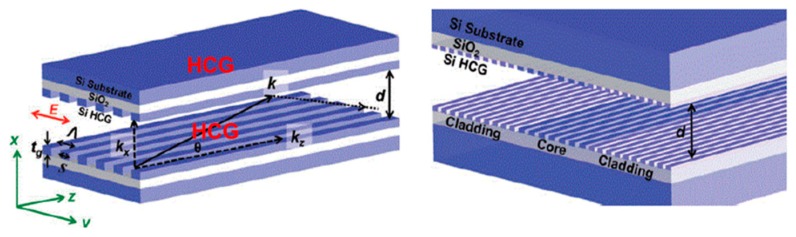
HCW with high contrast grating on a silicon substrate. Adapted with permission from [66], Copyrights 2011–2019 Walter de Gruyter GmbH.

Recently a novel HCW design was developed by Yang et al. [66] made from two planar, parallel, silicon-on-insulator wafers with subwavelength gratings for IR applications. The design shown in Figure 10 has a distinct advantage of efficiently guiding light (optical losses 0.37 dB/cm) without sidewalls for a 9 µm waveguide length, which allows the inflow and outflow of gases from the side. This design is suitable for microdevice to achieve a good interaction between the radiation and gas molecules. 

## 5. UV Photodetectors

### 5.1. General Overview

**Figure 11:**
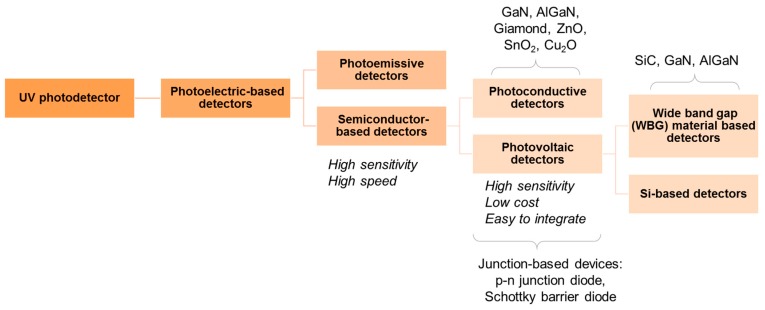
Classification of UV photodetectors.

**Figure 12:**
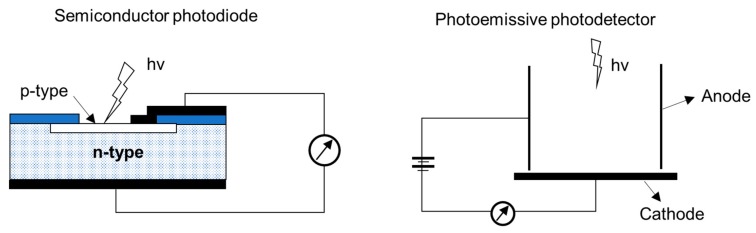
Principle of operation of the semiconductor-based photodetector and photo-emissive photodetector.

An ideal UV photodetector features high sensitivity, excellent photocurrent, or voltage linearity with incident optical power, high spectral selectivity with excellent visible rejection, good quantum efficiency, low noise (dark current), and long life (low UV ageing). Most of the sensors available for UV applications are based on the photoelectric effect, where material absorbs light and emits electrons. The different UV detector types and their classification are summarized in Figure 11 [67,68]. In photoemissive UV detectors, a solid surface emits an electron into a vacuum upon striking by photon as illustrated in Figure 12. A photomultiplier tube (PMT) is an example of a photoemissive detector. Primary photoelectrons are multiplied into secondary electron emission to produce a large cloud or gain of electrons [67]. In a semiconductor-based photodetector, the photon generates an electron-hole pair which are separated by an electric field as represented in Figure 12. The photon excites the electrons into the conduction band of the semiconductor. In case of the photovoltaic detector, the electron-pair is separated by the electric field of p-n junction or Schottky barrier, which leads to an external photocurrent proportional to the number of striking photons. In this type of detectors, the incident light causes a voltage to appear at the p-n junction while in a photoconductive detector, the incident light changes the internal resistance. The photovoltaic detector is commonly used for UV detection and is classified into Schottky barrier type, metal-semiconductor-metal (MSM) type, o-n junction type, and p-i-n junction type. The advantage and disadvantages of different kinds of photovoltaic detector are summarized in Table 3 [67,68,69,70]. For detail of each type of detector, the interested reader can refer to review articles published by Razeghi et al. [67], Shi et al. [68], Zou et al. [69], and Monroy et al. [70].

**Figure 13:**
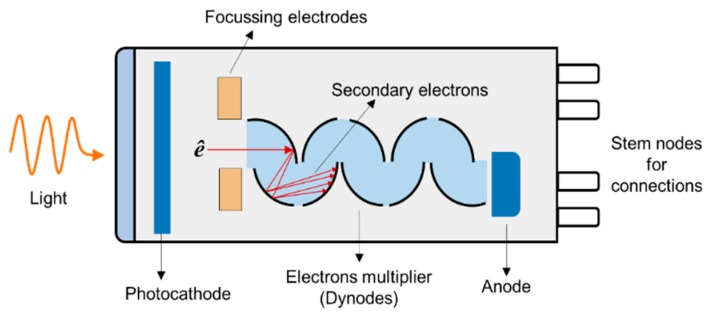
Schematics of the photomultiplier tube.

A PMT is a versatile, sensitive, and ultra-fast response device. It has a large detection area and can detect low-intensity levels of light. A typical PMT is made of a photo-emissive cathode (photocathode), focusing electrodes, an electrons multiplier, and electron collector (anode) enclosed in a vacuum chamber, as shown in Figure 13. The photoelectrons generated in the vacuum are accelerated and focused into dynodes. The electrons are then multiplied by the emission of secondary electrons by the successive dynodes until detected by the anode. The major disadvantages of PMT are low quantum efficiency and ageing effects.

A photodetector is usually selected according to the UV source, cut-off wavelength., and the design of optical and fluidic connections. The cut-off wavelength is the longest wavelength that the detector can detect. Dark current is critical in selecting a detector as it deteriorates the output signal-to-noise ratio of the photodetector and is generated in the detector in the absence of the radiation. The following relationships are useful for selecting a photodetector according to the specific requirements. The photocurrent generated is given by

(8) $$I=\;\int_{\lambda 1}^{\lambda 2}A_{chip}S_{chip}\left(\lambda \right)\;E\left(\lambda \right)\;d\lambda$$

where *I* is the photocurrent in Amperes, *A_chip_* is an active area of the chip in *m^2^*, *S_chip_*(*λ*) is the chip spectral sensitivity in A-W^−1^-nm^−1^ at the given *λ*, and *E*(*λ*) is the spectral irradiance of the UV light source in W-m^−2^. Equation (8) can be written in a simple form by assuming a constant value of *S* and *E*.

(9) $$I=A_{chip}\;S_{chip}\left(\lambda \right)\;E\left(\lambda \right)$$

The values of *S* can be obtained from manufacturer datasheet, usually its value is 0.1 A/W for SiC photodiodes, while *E* depends on the source employed. The current generated is usually in the range of nano-Ampere and an amplifier like trans-impedance is needed to obtain a measurable signal.

### 5.2. Challenges and the Latest Advancement

**Figure 14:**
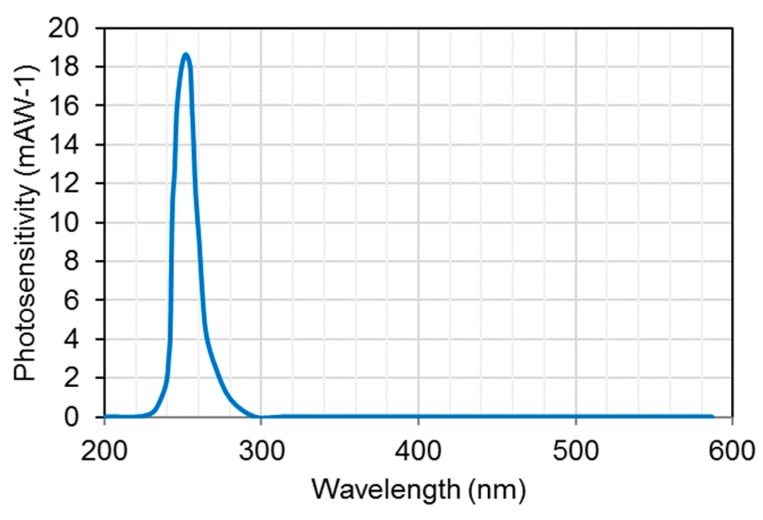
Photosensitivity spectrum of Si-photodiode (Data Source: Hamamatsu Photonics [25]).

Initially, narrow bandgap semiconductors were used for UV photodetectors, for example, silicon or III-V group compounds (for example gallium phosphide (GaP), gallium arsenide (GaAs)). For such materials, optical and interference filters were installed for spectral-range selectivity and to avoid material degradation [71]. These devices are sensitive to low-energy radiation and must be cooled for high-sensitive applications to minimize the effects of the dark current [67,68,69,70]. These devices also face an issue of ageing effect when exposed to radiation higher than their bandgap [72]. UV photodetectors based on wide bandgap (silicon carbide (SiC), gallium nitride (GaN), and aluminum gallium nitride (AlGaN)) work at room temperature and offer intrinsic blindness to visible wavelengths. They can operate even at high temperature and high power due to their high strength of chemical bonds and high thermal conductivity. There is no need of external filter to realize the desired UV spectrum even in the presence of high intense visible or infrared light. For example, Si-photodiodes are usually employed with an interference filter to achieve a monochromatic light with a narrow FWHM of 10 nm to minimize the effects of stray light. An example of a Si-photodiode photosensitivity spectrum is shown in Figure 14.

**Figure 15:**
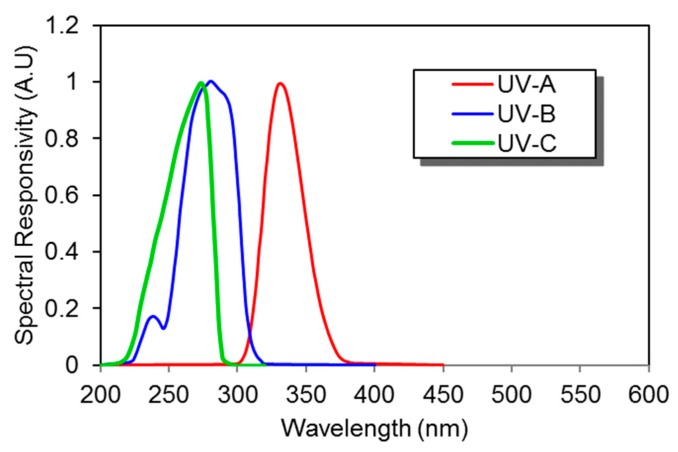
Spectral response of several silicon carbide (SiC) UV photodiodes (Data source: Sglux [73]).

SiC-based photodetectors are considered to be the most efficient UV photodetectors, for a wavelength range of 200 to 300 nm [72]. SiC-based photodiodes have visible-blindness (non-responsive towards visible range wavelengths i.e., 300 nm to 700 nm), low dark current, wide bandgap, high break down the electric field, fast response time, high thermal conductivity, and low thermal expansion. All these characteristics make them a suitable candidate for high temperature and radiation-resistant applications. Example of SiC-based photodiodes developed by Sglux exhibits relative narrow spectral response as shown in Figure 15.

## 6. Applications of Deep-UV Absorption Spectrophotometry

UV spectrophotometry has been applied for detection of several molecules, which are of industrial and domestic significance. For example, ozone, NO_2_, SO_2_, benzene, toluene, ethylbenzene, and xylenes show strong absorption in deep UV range as shown in Figure 1. Table 4 summarizes the airborne guidelines values of the above-mentioned species found in the literature. During the last few decades advancement in optoelectronics and optofluidics have led to the development of UV devices (i.e., LEDs and photodetectors) which are portable, high-sensitive, and cost-effective. Different approaches have been adapted to realize portable, sensitive, and selective sensors. In the following section, application of UV absorption spectrometry is discussed for detection of ozone, NO_2_, SO_2_, and BTEX. The different techniques and their analytical performance are compared by using sensitivity, limit of detection, time response, and the instrumentation used are summarized in Table 5.

### 6.1. Ozone

Ozone or trioxide (O_3_) is a significant atmospheric trace gas, which occurs in the troposphere and stratosphere. It is a toxic, unstable gas with strong oxidizing properties, and is colorless with a pungent odor. In the stratosphere, it protects the biosphere from harmful UV radiation from the sun while in troposphere it plays a vital role in atmospheric oxidation. In the lower troposphere, ozone is a pollutant and is detrimental to the ecosystem [74] and to human health [75]. In industry, it is used as an oxidizing agent in chemical reaction and bleaching applications. In the past, it was used as disinfection in the food industry or water purification. Ozone is also generated by the electric discharge in the air, which can be utilized as an indicator for the malfunctioning of electronic devices [76]. Different organizations, for instance, National Institute of Occupational Safety and Health (NIOSH) and Occupational Safety and Health Administration (OSHA) have established exposure limits of 0.1 ppm for ozone [77]. In order to detect ozone at such low concentration, different UV absorption spectroscopic approaches have been adapted by exploiting its absorption band in wavelength (245–320 nm) as displayed in Figure 1.

**Figure 16:**
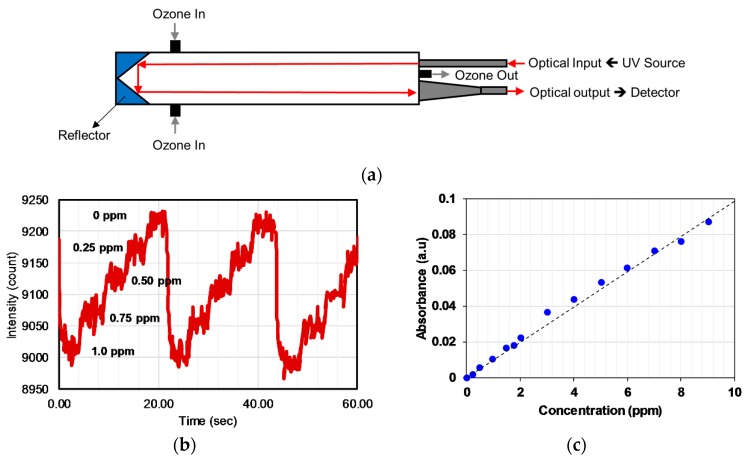
(**a**) Experimental setup with the optical source and detector at one side using a reflector on the other side. (**b**) Variation of the signal for 0.25 ppm change of ozone concentration. (**c**) Calibration curve obtained for different ozone concentrations in the range 0.2–10 ppm [76].

Maria et al. [76] developed an ozone monitor using a gas cell of length 400 mm with a retro-reflector to realize a longer optical path (~2 × 400 mm) by the back reflection of a light beam as shown in Figure 16a. A broadband UV source DH2000 (190–2500 nm) and spectrometer with filters were employed as a source and a detector, respectively, to achieve a peak wavelength of 300 nm with FWHM values of 40 nm. The UV source and detector were placed at one side and reflectometer on the opposite side. The linearity of 0.1–10 ppm with a limit of detection (LOD) of 0.1 ppm was achieved, as shown in Figure 16b,c. The LOD was limited by the reflectometer employed. 

**Figure 17:**
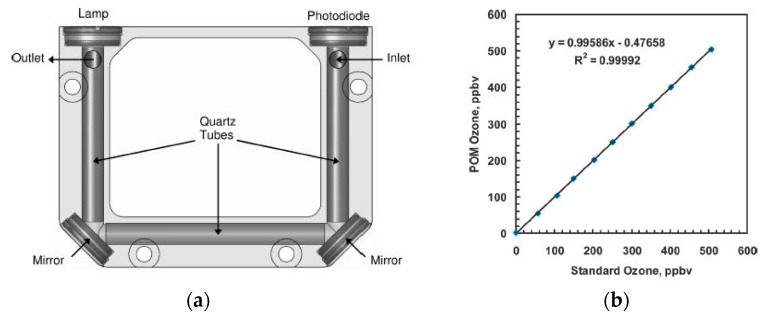
(**a**) The experimental setup with lamp and photodiode as a source and detector, respectively, with mirrors at the corners. Aluminum coated with Quartz was employed as a gas cell. (**b**) Calibration curve for different concentration of ozone. Adapted with permission from [59], copyright 2019 American Chemical Society.

Anderson et al. [59] developed a portable ozone monitor using aluminum coated with quartz as a hollow core waveguide of length 15 cm, as shown in Figure 17a. A low-pressure mercury lamp and photodiode (λ_center_ = 254 nm) with an interference filter were used as a source and detector, respectively. Ozone scrubber and Nafion tubes were installed as a filter, to avoid the noise of UV absorbing species and humidity, respectively. Good sensitivity with a precision of ≤2 ppb with a limit of detection (S/N = 3) of 4.5 ppb was reported. The sensor reported has good portability (size, 10 cm × 7.6 cm × 3.8 cm: weight, 0.3 kg) with a robust performance at different humidity, temperature, vibration, and physical orientation.

**Figure 18:**
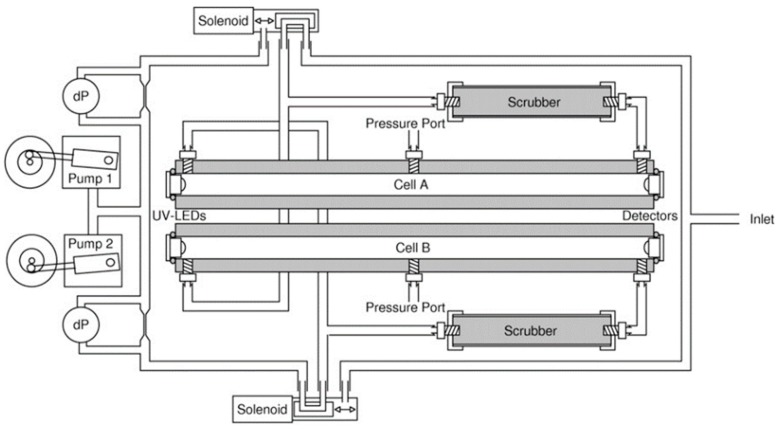
Experimental setup showing the optical and fluidics configuration of the ozone sensor [78].

An Ozone sensor was developed by Kalnajs and Avallone [78] for stratospheric ballooning applications. A UV LED (λ = 254 nm) was used as a source and integrated with a feedback photodiode and a thermistor to compensate for the variation of light intensity and temperature. Teflon tubes were installed as a gas cell with an optical path length of 48.8 cm and a diameter of 6 mm, as shown in Figure 18. SiC photodiodes were used as a detector. The effective area of the photodiode was increased by using a lens, from 1 to 11 mm^2^ without introducing extra noise and parasitic capacitance.

**Figure 19:**
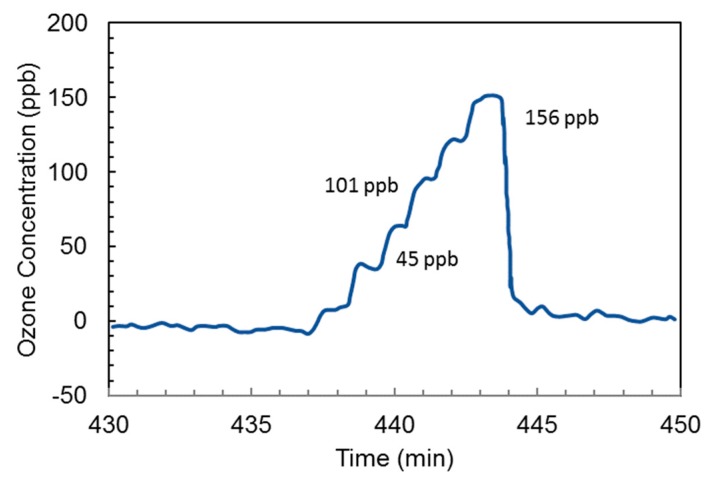
Ozone monitoring obtained during a calibration procedure [79].

Guiwen et al. [79] obtained an ozone detector with a resolution of 5 ppb for printing process applications. Ozone was detected with linearity and resolution of 0–156 ppb and 5 ppb, respectively, as shown in Figure 19. A mercury capillary lamp with a temperature control was employed using a quartz gas cell (length 28 cm) with photodiodes as a detector. The absorption distance/optical path length was increased by coating one side of the gas cell with reflective material to realize a two-way optical path. A second photodiode with a reduction lens was installed in front of a lamp and used as a reference measurement to avoid the emission drifts and to get a stable signal. The drift was decreased to 10 ppb per hour from 100 ppb per hour by matching the radiation intensity at the photodiode and the electronic circuit amplification.

**Figure 20:**
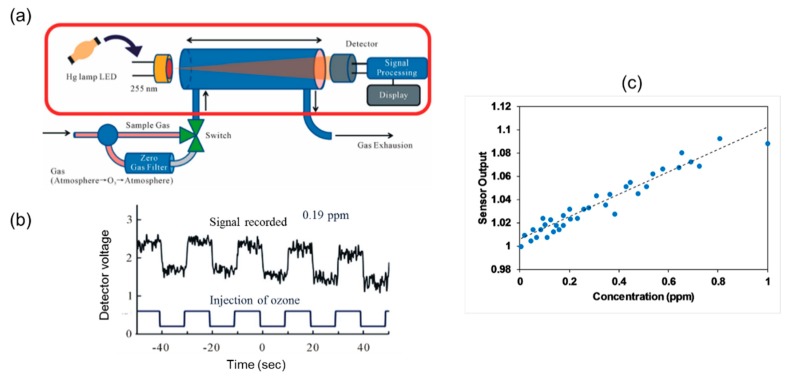
(**a**) Experimental setup for detection of ozone. (**b**) Signal for different flushing routine of ozone. (**c**) Calibration for different ozone concentrations up to 1 ppm [80].

Aoyagi et al. [80] developed an ozone sensor using LED (peak wavelength, 280 nm) and detected ozone down to 0.1 ppm with an accuracy of 0.5% using a gas cell of 20 cm as shown in Figure 20. LED was fabricated using AlGaN using coated with organic chemical vapor epitaxy crystal growth. 

### 6.2. Nitric Oxide (NO_2_) and Sulfur Dioxide (SO_2_)

NO_2_ is one of the harmful air pollutants and is reddish-brown with an acrid, pungent odor. The primary sources of generation are from fossil fuels [81], automobile exhausts, and by microorganism during the process of nitrogen fixation for agriculture fertilization [82]. Exposure to NO_2_ can cause inflammation of respiratory air tracts and can damage the lungs upon long exposure [83]. It plays a role in the formation of ground-level ozone, fine particulate matter, and acid rain [84]. The exposure limit of 5 ppm is defined by OSHA for NO_2_. SO_2_ is a colorless highly toxic gas with a strong irritating pungent odor. It is highly reactive gas create sulphuric acid when mixed with water, which is corrosive, can cause chemical burns and acid rains. OSHA designated the PEL for SO_2_ to be only 5 ppm. Its sources of generation are fossils fuels, automobile exhausts, boiler and refineries, and active volcanos [85]. It can harm eyes, lungs, and throat upon exposure [86].

**Figure 21:**
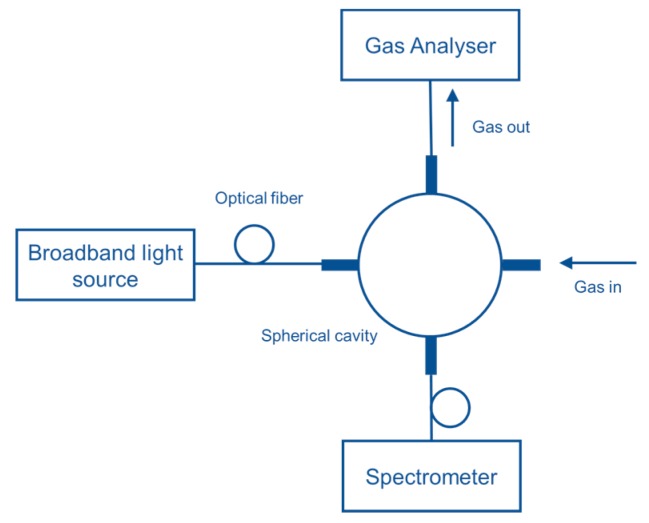
Experimental setup with the spherical gas cell for NO_2_ and SO_2_ detection [87].

**Figure 22:**
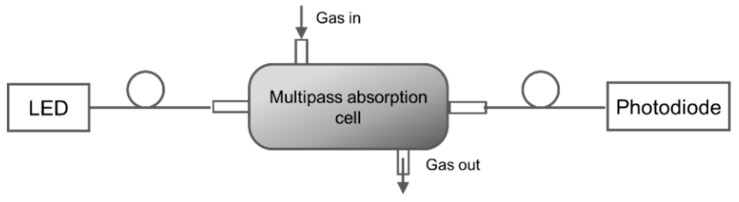
Experimental setup with a multiple-pass cell with LEDs and photodiodes [88].

Hawe et al. [87] developed a multipass absorption cell (spherical absorption cavity) for gas detection in visible and UV range. The gas cell was tested for NO_2_ and SO_2_ using broadband light as a source (Deuterium/halogen lamp) and spectrometer as a detector. The setup was also tested using LED and photodiode, respectively, for detecting NO_2_. A spherical gas cell with a diameter of 5 cm had an effective optical path length of 40–55 cm, was tested as represented in Figure 21. NO_2_ and SO_2_ down to 4 ppm and 11 ppm were detected with a response time of 4 s and 2 s, respectively. The same group [88] developed a LED-based sensor for SO_2_ and NO_2_ detection shown in Figure 22. Absorption gas cell and reflection gas cell with an optical path length of 20 cm and 8 cm, respectively, were tested.

**Figure 23:**
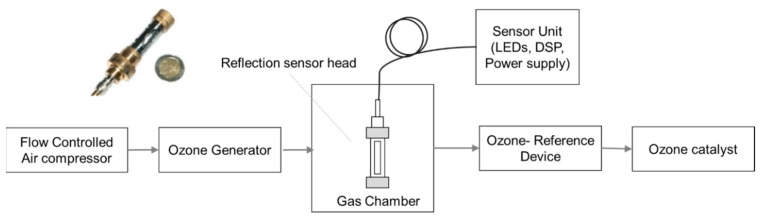
Setup for ozone measurement with ozone generator and ozone reference device. The sensor is shown at the left corner [89].

Based on absorption spectra of ozone given in Figure 1, LEDs with a peak wavelength of 255 nm, 285 nm, 320 nm, 405 nm, and 590 nm were used for detecting SO_2_ and NO_2_ with a high resolution of 1 ppm over a wide measurement range up to 1000 ppm and temporal dynamic range up to 10 ms. The setup was extended to detect ozone employing LED with a peak wavelength of 255 nm. The schematic of the setup is shown in Figure 23 [89]. Dynamic ranges of sub-ppb up to 10 ppm and 10 ppb to 100 ppm for absorption cell of 40 cm and 4 cm, respectively, has been reported.

### 6.3. BTEX

Benzene, toluene, ethylbenzene, and xylene (BTEX) are aromatic hydrocarbons and are some of the hazardous pollutants among VOCs. BTEX can be found in both indoor and outdoor environments and occur typically at high concentration in indoor spaces [90]. The common sources of generation of BTEX are coal burning, cigarette smoking, combustion and cleaning products, 3D printing, floor adhesives, paint, wood paneling, and traffic emissions [91,92,93]. Exposure to BTEX is considered one of the reasons for sick building syndrome [94]. Benzene is particularly toxic and acute occupational exposure to benzene can cause narcosis, headache, dizziness, drowsiness, confusion, tremor, and loss of consciousness [95,96,97]. The International Agency for Research on Cancer has identified benzene as carcinogenic to humans [98]. Exposure to toluene can influence the central nervous system, liver, kidney, and skin [99]. Exposure to xylene at a low level is associated with nervous system problems, fatigue, tremor, respiratory, kidney, and cardiovascular-related problems [100]. Due to the harmful nature of these molecules, there are stringent regulations for exposure limits to these gases. The established exposure limits for these air-borne pollutants by NIOSH and OSHA in the range from 100 ppb (for benzene) to 100 ppm (for toluene) as summarized in Table 4 [77].

**Figure 24:**
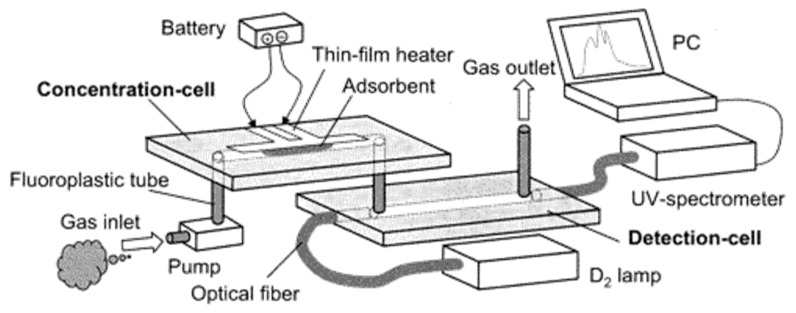
Experimental setup for detection of benzene, toluene, ethylbenzene, and xylene (BTEX). Detection cell was connected with pre-concentrator and UV-spectrometer. Adapted with permission from [101], copyright 2019 American Chemical Society.

**Figure 25:**
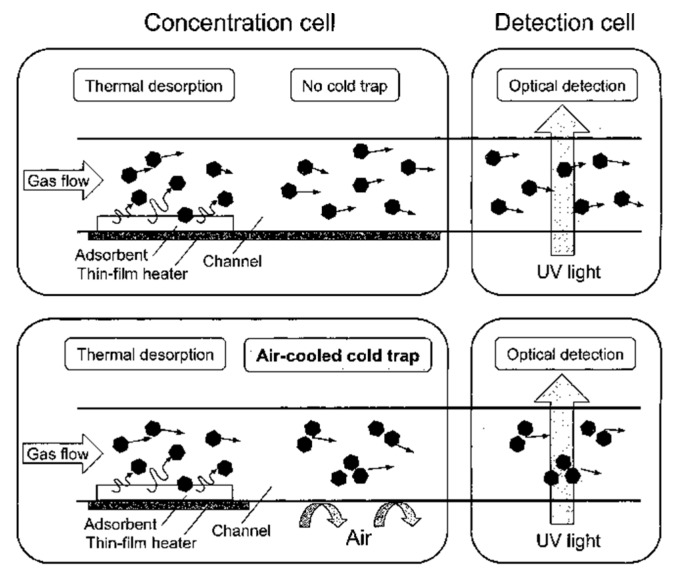
Pre-concentration cell with air-cooled air trap. Adapted with permission from [102], copyright 2019 American Chemical Society.

A synergic microfluidics approach was demonstrated by Ueno et al. [101] for BTEX detection using a UV absorption spectrometer with a pre-concentration unit. The pre-concentrator was composed of a microfluidic channel packed with an adsorbent, which traps BTEX molecules when air passes through and desorbs thermally (250 °C) using a heater at the bottom of the channel as shown in Figure 24. The collected molecules from the concentrator were analyzed in the optical cell, which was linked with a UV source (D_2_ lamp) and spectrometer. An aluminum gas cell with an effective optical path length of 20 mm was employed, and a significant 25-fold improvement was achieved. Detection limits of 4 ppm and 100 ppm were recorded for toluene with and without a pre-concentrator, respectively. The performance of the detector was enhanced by optimizing the geometry of the pre-concentrator and by inserting an air-cooled cold trap in-between the pre-concentrator and optical gas cell, as shown in Figure 25 [102]. The air-cooled cold trap maintains the molecules together and prevents the dilution of the desorbed molecules. An 80-fold reduction in limit of detection was reported, i.e., 0.05 ppm. The air-cooled traps were installed to prevent dilution of desorbed gas molecules. The structural change (wider channel) and cold trap contributed 4-fold and 20-fold reductions in limit of detection, respectively. BTEX molecules have close peak absorbance wavelengths, and the differentiation of the molecules is difficult. However, using µGC for separating each molecule before detection cell can help in identification of each molecule. 

Generally, gas sensor sensitivity can be enhanced by employing a concentration unit before the detection cell, often called pre-concentrator. The sample gas can be enriched by collecting the molecules from the carrier gas into the adsorbent over a period referred to as the concentration time. The collected molecules are then released instantaneously using a thermal heater. The release of molecules at a small volume and short time leads to 10^3^–10^5^ folds amplification of concentration level at the cost of longer response time [103,104,105].

**Figure 26:**
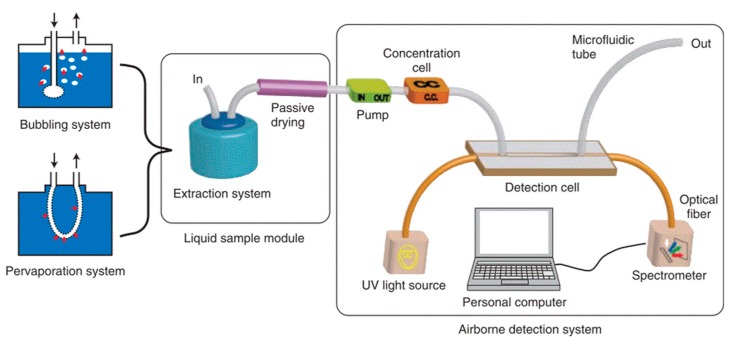
Setup for the detection of aqueous benzene [103].

A further improvement in performance was reported by replacing the detection cell with a platinum-coated cell (length, 2 cm) and the pre-concentrator packed with meso-silicates adsorbent [106]. The system demonstrated good sensitivity and linearity (10–100 ppb), and a limit of detection of 10 ppb was reported for benzene. The prototype was extended for the detection of aqueous BTX molecules [103]. Two intermediate modules were added for extraction from the aqueous phase to the gaseous phase and passive drying process as shown in Figure 26.

**Figure 27:**
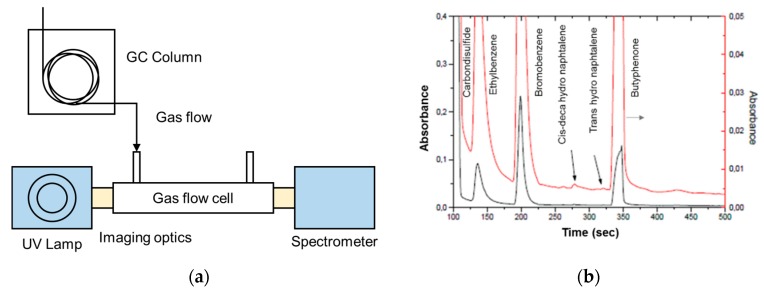
(**a**) Schematic of the experimental setup with Gas Chromatography. (**b**) Chromatograph obtained for different molecules (red line represents a magnified version (8 times) of the chromatogram) [108,109,110].

Eckhardth et al. [107] used an aluminum-coated HCW (length,1 m and diameter, 1 mm) with a 30 W deuterium lamp for analyzing the absorption spectrum of SO_2_. The UV absorption of SO_2_ was compared with its IR absorption. A CCD-based spectrometer was used with a spectral resolution of 0.01 nm in the wavelength range 175 to 210 nm. SO_2_ concentration of 1.1 ppm was first analyzed, and it was found that SO_2_ absorbs stronger (10 times approx.) in UV range compared to IR. The system was then tested to measure the absorbance of ammonia and nitric oxide of 1.1 ppm in the wavelength range of 185 to 205 nm [108,109,110]. The system sensitivity and selectivity were enhanced by coupling the prototype with GC (length 25 m, diameter 0.32 mm) as given in Figure 27a [108]. A mixture of compounds (Ethylbenzene, bromobenzene, cis-decahydronaphtalene, trans-decahydronaphtalene, Buthyrophenone, diphenylsulfoxide, carbon disulphide) with close retention time was tested, spectrum was recorded in the time domain for wavelength range 170–320 nm, and all the compounds were distinctively separated as represented in Figure 27b.

**Figure 28:**
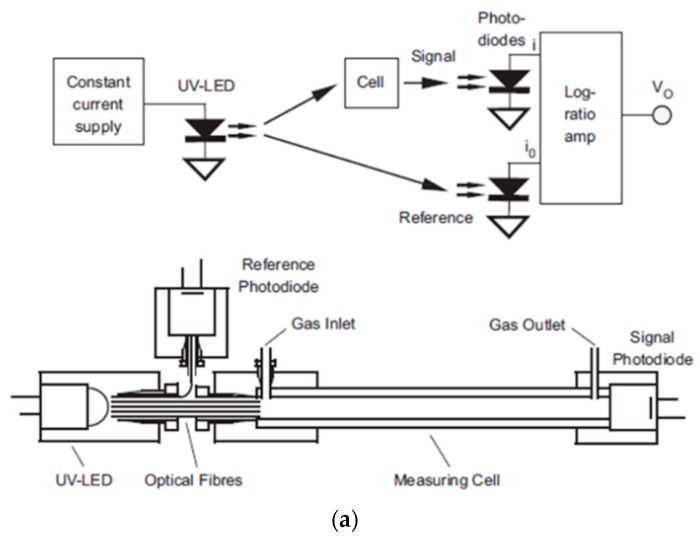

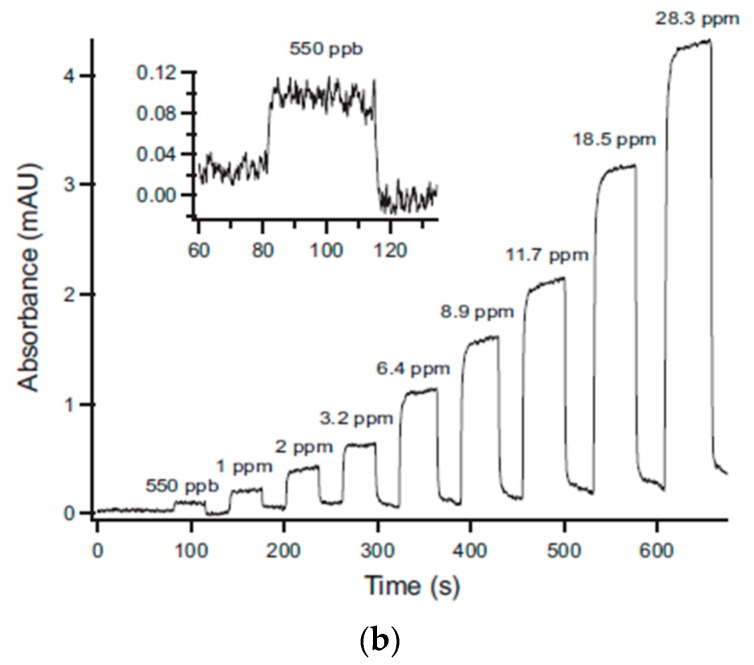
(**a**) Experimental setup using photodiodes for a measurement and a reference signal. (**b**) Variation of absorbance with the concentration of gaseous toluene. Adapted with permission from [111], copyrights 2019 Elsevier.

Bui et al. [111] developed a self-referenced photometer using a UV LED and photodiodes for the direct detection of BTEX molecules. Aluminum HCW (length, 40 cm and Inner diameter, 2 mm) was employed as a gas cell with UV LED (λ, 260 nm) and photodiodes coupled via optical fiber. The absorption values were directly recorded by using integrated circuit log-ratio amplifier converting the reference and test photocurrent into voltage values, as shown in Figure 28a. A sensitivity of 235 µAU/ppm (LOD, 0.5 ppm) and 62 µAU/ppm was recorded for p-xylene and benzene, respectively, with excellent reproducibility (relative standard deviation < 2.3%) and linearity in the range 0.5–110 ppm (R^2^ = 0.999).

**Figure 29:**
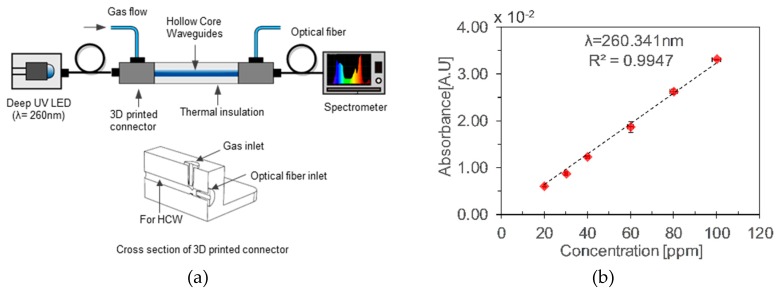
(**a**) Experimental setup with aluminum HCWs. (**b**) Calibration curve for different concentration of gaseous toluene [24].

Khan et al. [24] demonstrated a simple toluene detector using a glass and aluminum-based gas cells. A fiber-coupled deep UV-LED was used as a source and a mini-spectrometer as a detector with 3D printed connectors, as shown in Figure 29a. The performance of the two types of HCWs was investigated and compared. A limit of detection of 8.15 ppm and 12.45 ppm was reported, with good repeatability and linearity, as shown in Figure 29b.

## 7. Conclusions and Outlook

There are several molecules of environmental and domestic significance, which show strong deep-UV absorption. This intrinsic property can be exploited for the development of a gas sensor using absorbance measurement at a specific wavelengths range. UV absorption spectrophotometry provides a sensitive, reliable, self-referenced, and selective approach for gas sensors development. Recently, portable and efficient UV optoelectronic and optofluidics components have been developed, for example LEDs, HCWs, and photodiodes. These portable devices can be utilized to develop a portable deep-UV absorption spectrophotometer, which can rival the analytical performance of a lab-based deep-UV absorption spectrophotometer.

LEDs offer a stable, efficient, portable, and a narrow emission-band UV source for analytical applications with ease of alignment, low cost, and enhanced lifetime. The challenges faced by deep-UV LEDs are the need for a highly stable power supply source, low power emission, and their susceptibility to thermal fluctuation. Advances in nitride semiconductors have pushed LEDs into the UV-C band with improved power (1.5 mW) and low parasitic emission, however thermal-induced noise issue is still present. A constant power supply, a well-designed thermal management, and self-reference scheme can minimize this noise and can enhance the stability and intensity of the output signal. Aluminum-based HCWs are an attractive option to be employed as a gas cell due to their efficient UV transmission, ease of alignment, and fabrication process. However, size of the gas cell is a bottleneck for a portable UV spectrophotometer. Recently, the newly developed i-HCWs can be used as a gas cell, which has a smaller footprint area with a longer optical path, easier integration, and simpler fabrication process. Such i-HCW have not been reported for deep-UV application, however the design has a tremendous potential to be used as a gas cell with a longer optical path on a smaller footprint area for improved sensitivity and portability. The desired features of UV photodetectors for analytical applications are spectral selectivity with visible rejection, minimal dark current, linearity, and resistance against UV-aging. Among the different UV photodetectors, SiC photodiodes have shown excellent performances for a narrow beam of UV radiations (FWHM ~10 nm) without the use of any spatial filters and can be easily coupled via optical with other components. A well-designed UV spectrophotometer based on LEDs, HCWs, and photodiodes can rival the analytical performance of detectors based on conventional UV lamps and commercial spectrometers. 

This review covers the latest advancement in the domain of UV absorption spectrophotometry and its application for gas sensing. New developments are emerging in the domain of optoelectronics, optofluidics, and material science, which would push the frontiers of this multidisciplinary area. This study provides useful guidelines for deep UV absorption spectrophotometry that are not only applicable for gas sensing, but also for analytes detection in liquid media, for instance, HPLC.

## Figures and Tables

**Table 1:** Comparison of different gas detection techniques [12,14,15].

Method/Technique	Advantages	Limitations
Metal Oxide Semiconductor	Good sensitivity. Low cost. Quick Time response. Easily to integrate.	Relative low selectivity. High-operating temperature. Zero-drift and ageing effect. Mostly affected by humidity and temperature.
Electro-Chemical	Relative sensitive. Reliable. Low power consumption. Quick time response. Lower cost	Zero drift. Aging, which leads to a shorter life.
PID	High sensitivity to aromatics. Quick response. Portable.	Low selectivity, all the gases with IP equal or lower the photon may be detected (total VOC concentration). Cost.
Piezoelectric	Good sensitivity. Portable. Good dynamic range.	Large measurement noise. Weak selectivity. Zero drift and cross-reactivity. Interference from humidity and temperature.
Optical sensors	Excellent sensitivity. High gas specificity. Minimal drift and cross-response. Non-destructive. Ultra-fast response.	Size High cost

**Table 2:** Comparison of UV sources for portable spectrophotometry.

	LED	Deuterium Lamp	Xenon Flash Lamp	Mercury Lamp
Wavelength	Single peak	Relatively wide spectrum 120–400 nm	Broad-spectrum 160–2000 nm	Broad-spectrum 185–2000 nm
Stability of light output	Excellent temporal and spatial stability.	Good. Fluctuation <0.005%	Relatively poor. Fluctuation <3%	Relatively poor. Fluctuation <2%
Warm-up time	Instantaneous	20–30 min	Instantaneous	1–15 min
Life (hours)	3000–10,000	2000–4000	400–5000	500–3000
Input wattage (W)	DC powered 6–10 V	5–150	2–60	50–500
Thermal effect on samples	None. LEDs do not emit forward heat	Sample can be affected by the heat from the lamp	None	Sample can be affected by the heat from the lamp
Cost	Low	High	High	Low
Drive electronics	Simple	Complex	Complex	Complex
Safety	Low voltage and cold light source	High power supply (Input wattage 5–150 W) and hot lamp surface	High voltage supply (Input wattage 2–300 W): sparking risk	High voltage supply (Input wattage 50–500 W) and contains mercury in fragile quartz envelop

**Table 3:** Advantages and disadvantages of photovoltaic UV detectors [67,68,69,70].

Photodetector	Advantages	Disadvantages
Photoconductor	Simple design, easy process control, high gain	Large dark current, slow time response
Schottky UV detector	Low dark current, quick time response, high sensitivity, and quantum efficiency	Higher absorption losses, shallow-semiconductor contact
p-n and p-i-n detectors	Fast time response, high impedance, low dark current, low bias operation, high-frequency operation, easier fabrication	The response is dependent on the dopant used which impairs the spectral response
Metal-semiconductor-metal (MSM)	Fast time response, minimally affected by bias, simple fabrication process, low cost, easy integration	Lower gain and spectral response

**Table 4:** Different exposure limits recommended by different organization.

Molecules	NIOSH-Recommended Exposure Limit (ppm) ^a^	OSHA -Permissible Exposure Limit (ppm) ^b^	ACGIH-Threshold Limit Value (ppm) ^c^	ANSES (VGAI) France-Long Exposure (ppm) ^d^
**Ozone**	0.1	0.1	0.05–0.2 ^e^	-
**NO_2_**	1	5	0.2	-
**SO_2_**	2 [TWA]	5	0.25	-
**Benzene**	0.1	1	0.5	0.0006
**Toluene**	100	200	20	5.31
**Ethylbenzene**	10	10	10	0.345
**Xylene(m-,o-,p-)**	100	100	100	-

^a^ National Institute of Occupational Safety and Health (NIOSH)-recommended exposure limit is an exposure for 8 or 10-h time weighted-average(TWA); ^b^ Occupational Safety and Health Administration (OSHA) permissible exposure limit are expressed as a time-weighted average; the concentration of a substance to which most workers can be exposed without adverse effect averaged over a normal 8-h workday or a 40-h workweek; ^c^ American Conference of Governmental and Industrial Hygienists (ACGIH) threshold limit value are expressed as a time-weighted average; the concentration of a substance to which most workers can be exposed without adverse effects; ^d^ National Agency for Food Safety, Environment and Labor (ANSES) Interior Air Quality Guide Values (VGAI) France; ^e^ Depend on time and workload.

**Table 5:** Comparison of different deep-UV absorption spectrometry for ozone, NO_2_, SO_2_, and BTEX.

S. No.	Molecules Detected	Source	Peak Wave Length	Detector	Gas Cell (Materials)	Optical Path Length	Characterization				Remarks	Ref.
							Limit of Detection	Sensitivity (μAU/ppm)	Linearity	Time Response		
1	BTEX	Deep UV LED	260 nm	Photodiode	Aluminum	40 cm	Benzene = 1.2 ppm Toluene = 658 ppb Ethyl-Benzene = 612 ppb O-Xylene = 600 ppb m-Xylene = 607 ppb p-Xylene = 457 ppb	Benzene -62 Toluene -152 Ethylbenzene-166 Xylene(-o)-185 m-Xylene(-m)-169 p-Xylene(-p) -235	934 ppb-60 ppm	fast	Good reproducibility of RSD 2%. Carrier gas: N_2_	[111]
2	Toluene	Deep UV LED	260 nm	spectrometer	Aluminum and glass HCW with aluminum coatings	25 cm	8.1 ppm	200	10–100 ppm	-	Good RSD 2.5%. Carrier gas: N_2_	[24]
3	SO_2_, NO_2_, Ammonia, Ethyl benzene, bromobenzene, cis-decahydronaphtalene, trans-decahydronaphtalene, Buthyrophenone, diphenylsulfoxide, carbon disulphide	Deuterium lamp	175–210 nm	spectrometer	Aluminum-coated silica HCW	1 m	1.1 ppm of SO_2_ was analyzed	-	-	-	UV and IR absorption were compared. Carrier gas: N_2_	[107] [110] [108]
4	BTX	UV D_2_ lamp	230–270 nm	UV spectrometer	Pyrex wafer with Platinum coating	2 cm	4 ppm for toluene	-	-	20 sec for detection cell (The total analysis time is different and depend on the pre- concentration time)	Microfluidics-based device. Pre-concentrator enhanced the LOD from 4 ppm to 100 ppm for toluene. Carrier gas: N_2_	[101]
5	BTX	UV D_2_ lamp	230–270 nm	UV spectrometer	Pyrex wafer with the Platinum coating	2 cm	0.05 ppm for toluene	-	-	Sampling time 30 min	Air-cooled traps were placed to avoid adsorbed gases dilution. Carrier gas: N_2_	[102]
6	BTX	UV D_2_ lamp	230–270 nm	UV spectrometer	Channel in glass substrate with platinum coating	2 cm	10 ppb for benzene	-	10–100 ppb	50 min total sampling time	Several parameters were optimized to enhance the LOD to 10 ppb. Carrier gas: N_2_	[106]
7	Ozone	Deuterium lamp	Wavelength range	Spectrometer with filter	PTFE (Polytetrafluoroethylene)	40 cm		0.1 ppm	0.1–10 ppm		The optical path was increased by using a reflector on one side. Carrier gas: Air	[76]
8	Ozone	Low -pressure Hg lamp	255 nm	Photodiode with an interference filter	Aluminum with Quartz lining	15 cm	Precision is less than 2 ppb LOD 4.5 ppb	-	-	10 s	The device is small in size with low power consumption. Carrier gas: Air. Nafion tubes were installed to remove humidity from the air.	[59]
9	Ozone	LED	280 nm	AlGaN detector	-	20 cm	0.1 ppm	-	0–1 ppm form plot	-	Photodiodes were discussed in detail.	[80]
10	Ozone	LED	254 nm	SiC photdiodes	Teflon tubes	48.8 cm	-	-	-	-	Optoelectronics and data acquisition were discussed in detail	[78]
11	Ozone	LED	255 nm	photodiode	Aluminum	40 cm and 4 cm	Sub ppb to 100 ppm	-	-	-	- Carrier gas: Air	[89]
12	NO_2_, SO_2_	Deuterium/halogen lamp and LED		Spectrometer and photodiode	Multi-pass spherical gas absorption	40–50 cm (Effective optical path length)	4 ppm NO_2_ 11 ppm SO_2_	NA	0–50 ppm	2–4 sec	Integrating sphere (multi-pass gas cell) was tested. Carrier gas: N_2_	[87]
13	NO_2_, SO_2_	LED	255 nm, 285 nm, 320 nm, 405 nm and 590 nm	photodiode	Aluminum	20 cm and 8 cm		Resolution 1 ppm	Up to 100 ppm	10 ms	Carrier gas: N_2_	[88]
